# Quantitative Nanometrology of Binary Particle Systems Using Fluorescence Recovery after Photobleaching: Application to Colloidal Silica

**DOI:** 10.1021/acs.langmuir.5c01287

**Published:** 2025-05-19

**Authors:** Daniel Doveiko, Lisa Asciak, Simon Stebbing, Wenmiao Shu, Karina Kubiak-Ossowska, David J. S. Birch, Yu Chen

**Affiliations:** † Photophysics Group, Department of Physics, 3527 University of Strathclyde, Glasgow G4 0NG, U.K.; ‡ Department of Biomedical Engineering, University of Strathclyde, Glasgow G4 0NW, U.K.; § 41945 PQ Silicas UK Limited, Warrington WA5 1AB, U.K.; ∥ ARCHIE-WeSt, Department of Physics, University of Strathclyde, Glasgow G4 0NG, U.K.

## Abstract

We present an application of fluorescence recovery after photobleaching (FRAP) to measure the size of the individual nanoparticles in binary systems. The presence of nanoparticles with varying sizes was successfully demonstrated using a straightforward biexponential model and their sizes were accurately determined. Furthermore, we have demonstrated the benefits of preprocessing the data using a simple machine learning algorithm based on the gradient boosting machine and fitting the resulting curves to a triexponential model. This approach allows the accurate recovery of the sizes of each of the three components in a binary particle system, namely, the 6 nm LUDOX HS40, 11 nm LUDOX AS40, and the free R6G labeling dye. Lastly, it has been demonstrated using molecular dynamics simulations that R6G adsorption to silica nanoparticles (SNPs) is indeed size-dependent, with larger constructs as the preferred target because of their higher charge and smaller curvature. The theoretical and experimental results were therefore consistent with one another.

## Graphical abstract

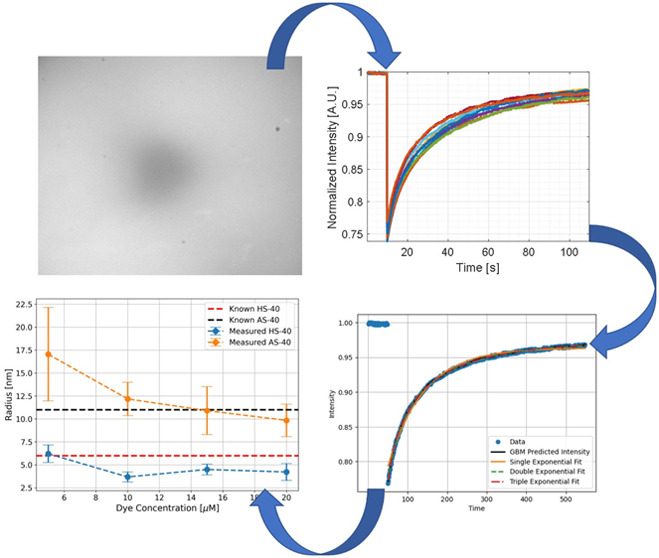

## Introduction

Nanotechnology spans scientific fields such as chemistry, physics or material science and its impact on the 21st century can be compared with molecular biology, semiconductor technology and other similar breakthroughs of the 20th century.^1^ Nonetheless, with the growth of nanotechnology, there is an ever-growing need to find efficient ways to characterize nanoparticles (NPs). Typically, methods such as transmission electron microscopy (TEM), small-angle X-ray scattering (SAXS), and dynamic light scattering (DLS) dominate the field, but they can be expensive, require complex sample preparation, have problems dealing with polydispersed systems or are inaccurate for particles under 10 nm in size, thus vastly limiting their usability.^2–4^ As a result, finding a suitable alternative that could successfully characterize polydispersed systems with particles below 10 nm radius is of significant importance and interest. We have recently shown that time-resolved fluorescence anisotropy can be used successfully as a nanometrology tool by providing accurate size estimates of colloidal silica nanoparticles (SNPs) and sodium silicates.^5–7^ However, only average particle size can be measured by this method with no insight of the size distribution. An alternative method for measuring particle sizes is fluorescence recovery after photobleaching (FRAP). FRAP’s main benefit is that it can be performed in almost every laboratory equipped with a modern fluorescence microscope.

Over the last few decades, FRAP has established itself as one of the most widely used methods to study mass transport and diffusion in biological molecules. With the rise of commercially available confocal laser scanning microscopes (CLSMs) in the second half of the 1990s and the introduction of green fluorescent protein^8,9^ FRAP has become an indispensable tool in biochemical laboratories around the globe.^10^ A fluorophore is irreversibly photobleached in FRAP experiments by a short laser pulse. Next, bleached molecules in the affected area are replaced with fluorescent molecules that diffuse into this space and the consequent fluorescence recovery in the bleached area is monitored as a function of time.^11^ In the case when the probe can diffuse freely, the recovery curves allow the diffusion coefficient and resulting hydrodynamic radius (*R*_h_) of the fluorescent molecules to be determined using the Stokes–Einstein relationship.^12^ Due to the nature of the measurement, the particles of interest must have intrinsic fluorescence, or be labeled with a fluorophore which can be photobleached without altering the structure of the particles of interest. Additionally, the sample must be of a certain transparency at the specific laser wavelength to allow the laser to penetrate the medium. An important advantage of FRAP over other techniques such as diffusion NMR^13,14^ is its capability to probe diffusion at the micrometre scale, which introduces spatial resolution to the measurements. Nonetheless, the main application fields of FRAP are still centered around biophysics and biochemistry^15^ involving proteins and cells^16–19^ and its usage in nanometrology is limited. This is because it is common for multiple equally well-fitting models to report conflicting kinetics.^20,21^ There has been some recent development in the field of FRAP in colloidal science, involving emulsions,^22,23^ gels^24^ or liquid crystals.^25^ Pihl et al. developed FRAP-responsive probes using fluorescent silica nanoparticles as diffusion probes for mass transport measurement.^26^ Nonetheless, in all of the cases mentioned above, the studied systems had either large particles/high viscosities, or involved synthesized fluorescent particles.

In this work, we successfully applied FRAP to LUDOX colloids without their modification by simply mixing them with Rhodamine 6G (R6G). The LUDOX grades studied were the 6 nm radius HS40 and 11 nm radius AS40. We have demonstrated that FRAP can effectively measure individual colloidal particle sizes using a straightforward multiexponential model and, more importantly, distinguish different nanoparticle sizes in a mixture. This approach provides a valuable alternative to more complex and less accessible techniques like SAXS or TEM. Furthermore, we extended our integration of fluorescence measurements with Molecular Dynamics (MD) simulations, reinforcing our experimental findings. Our MD simulation confirmed that labeling silica nanoparticles (SNP) with R6G has minimal impact on the measured size.^27^ MD results also revealed that R6G preferentially adsorbs better to larger SNPs, which exhibit a higher net negative charge and lower curvature.

## Materials and Methods

### Chemicals and Reagents

The LUDOX colloids and R6G (Bioreagent, suitable for fluorescence) used in this work were purchased from Sigma-Aldrich. According to the product description, LUDOX HS40 contains amorphous particles with a nominal radius of 6 nm, 45 cP viscosity at 25 °C, pH of 9.5 and is stabilized with a sodium counterion, while LUDOX AS40 contains amorphous particles with a 11 nm radius, viscosity of 20 cP at 25 °C, pH of 9.2 and is stabilized with ammonium hydroxide.

### FRAP Measurements

As the nanoparticles present in the colloids are nonfluorescent, they were labeled with Rhodamine 6G (R6G) with the label concentration ranging from 5 to 20 μM. To perform the measurements, 7 μL of labeled colloid were placed on a microscope slide with a 0.12 mm spacer and covered with a coverslip. After that 50 prebleach frames were taken at 0.2 s time step followed by 3 s bleach at full laser power over a 10 μm radius region of interest (ROI) and another 500 frames with 0.2 s step during the postbleach phase. Ten measurements were taken at different locations on the sample, ensuring sufficient distance between them to prevent interference from bleached molecules in previous experiments. Next, the data was corrected for the residual bleaching from the LEDs during the imaging by monitoring the intensity as a function of time in an ROI located further from the bleaching area. All measurements were performed on a Cairn Research Open Frame microscope, utilizing 530 nm LEDs for imaging (Cairn Research) and 150 mW CW 532 nm laser for bleaching (Coherent) and imaged using CellCam Kikker camera (Cairn Research), through an Olympus UPLXAPO 100× Oil Immersion Objective with a numerical aperture of 1.45. The preliminary image analysis was performed using ImageJ,^28,29^ while the extracted data was analyzed and fitted using MATLAB and Python.^30,31^

### FRAP Data Analysis

The optimal parameters for the fitting of the data obtained for the pure colloids were recovered by deploying a two-step logarithmic search grid. First, a search over the larger range of parameters using a coarse grid was performed, followed by a search over the predicted area using a finer grid. As typical in FRAP experiments, the data was fitted to mono and biexponential models with the latter one proving to be more suitable.

For the binary particle systems, the two-step logarithmic grid search method was found to be no longer efficient due to its relatively high computational cost and the direct fitting was not reporting satisfying results. Therefore, a custom fitting routine based on machine learning (ML), namely the gradient boosting machine (GBM) algorithm was used.^32,33^ The algorithm builds an ensemble of weak prediction models by using the experimental data and sequentially adds models that correct errors made by previous ones with the key idea being the minimization of a loss function by iteratively training models to fit the residuals of the combined model at each step. GBM builds models iteratively and focuses on improving predictions for challenging cases rather than being overly influenced by extreme values. This means that outliers have less impact on the overall model performance than many traditional methods as GBM progressively adjusts to reduce their effect, allowing for more reliable and stable predictions even in noisy data. As a result the obtained data were first preprocessed using the ML algorithm followed by a fitting of it to a triexponential model where each component corresponds to different system components, namely the shortest recovery half-time corresponds to the free R6G, the middle component corresponds to the HS-40 with a nominal radius of 6 nm and the longest component is attributed to the AS-40 particle with a radius of 11 nm. The resulting method proved to be significantly faster than a two-step logarithmic grid search over a large range and allows for future development in improving the fitting precision by incorporating larger data sets. The algorithm used the full ten repetitions for each sample, plus another five synthetic curves generated using bootstrapping to smooth out the data. In all cases the corrected FRAP recovery curves were fitted to a multiexponential model

1 $$I\left(t\right)=b+\sum_{i=1}^{n}A_{i}\left(1-\exp\left(-\frac{t}{\tau_{i}}\right)\right)$$

where τ_*i*_ are recovery half times and *A*_*i*_ are the corresponding pre-exponential factors. To calculate the diffusion coefficient, the following relation was used

2 $$D=\Gamma \frac{\omega^{2}}{4\tau }$$

where Γ is the correction factor for the Gaussian beam shape equal to 0.88, ω is the radius of the bleaching spot and τ is the recovery half-time. Next, the particle hydrodynamic radius *R*_H_ was calculated using the Stokes–Einstein relation

3 $$R_{H}=\frac{k_{B}T}{6\pi \eta D}$$

where *k*_B_ is the Boltzmann constant, *T* is the temperature equal to 293.15 K and η is the sample viscosity measured using the rotational rheometer.

### Rheological measurements

Rheological measurements were performed on a Kinexus Prime pro+ (Netzsch) rotational rheometer using a 40 mm diameter cone with a 4° cone angle. For the measurement, 1.19 mL of sample was deposited between the plate and a cone, which was followed by a sample cooling until 20 °C temperature was reached and 5 min equilibration at the designated temperature. Next, viscosity measurements were performed in the range of 0.1 to 100 s^–1^ shear rates with 10 points per decade and a ramp time of 30 s. In all cases, the steady state was reached, signaling that the precise value of the viscosity was obtained. All data were analyzed using Python.

### Molecular Dynamics Protocol

The CHARMM-GUI^34^ server was used to create the SNPs and design the preliminary systems. Namely, the SNPs were created using the Nanomaterial Modeler^35^ extension of CHARMM-GUI, while the R6G was based on the B3LYP/6–31G** parametrized structure reported by Chuichay et al.^36^ and Vaiana et al.^37^ and has been successfully used in our previous MD simulations.^27^ To match the experimental conditions, the pH of the system was set to 9 through the deprotonation of the surface silanol groups of the SNPs. As simulating SNPs at their real sizes (6 and 11 nm radii) would be computationally expensive, the system was rescaled. Namely, the 6 nm radius HS40 SNP was modeled as 25.7 Å, while the 11 nm AS40 was modeled as 51.3 Å radius SNP. This allowed maintaining the SNP size ratio of around 2, while significantly reducing the computational costs. However, it is essential to point out that the system rescale artificially suppresses the dye-to-SNP adsorption arising from the increased SNP diffusivity and larger curvature. As a result, the observed simulation mechanics should act as a guide by indicating the trends rather than exactly replicating the experimental conditions. The system had one large SNP and three small SNPs in all cases, with the dye molecule number ranging from one to four. Next, the obtained .psf, .crd, parameter, and topology files for all system components were uploaded to the Multicomponent Assembler^38^ of CHARMM-GUI to combine all the components in a vacuum. The obtained multicomponent system was solvated in VMD^39^ with TIP3P^40^ water model and neutralized using NaCl. One Cl^–^ ion was needed per single R6G to neutralize its cationic charge, while all of the Na^+^ ions present in the system came from the ionization of the SNPs as to the desired pH. Due to varying R6G concentrations, the above resulted in four simulation systems, each containing a single 51.3 Å radius SNP surrounded by three 25.7 Å SNPs, and 1, 2, 3, or 4 dye molecules. The R6G molecules were positioned to ensure equal probability of adsorption onto any SNP, avoiding bias toward a specific one with the minimal distance between each of the system components being no less than 15 Å. The total number of atoms per system was around 450,000. The initial system setup is shown in Figure 1.

**Figure 1:**
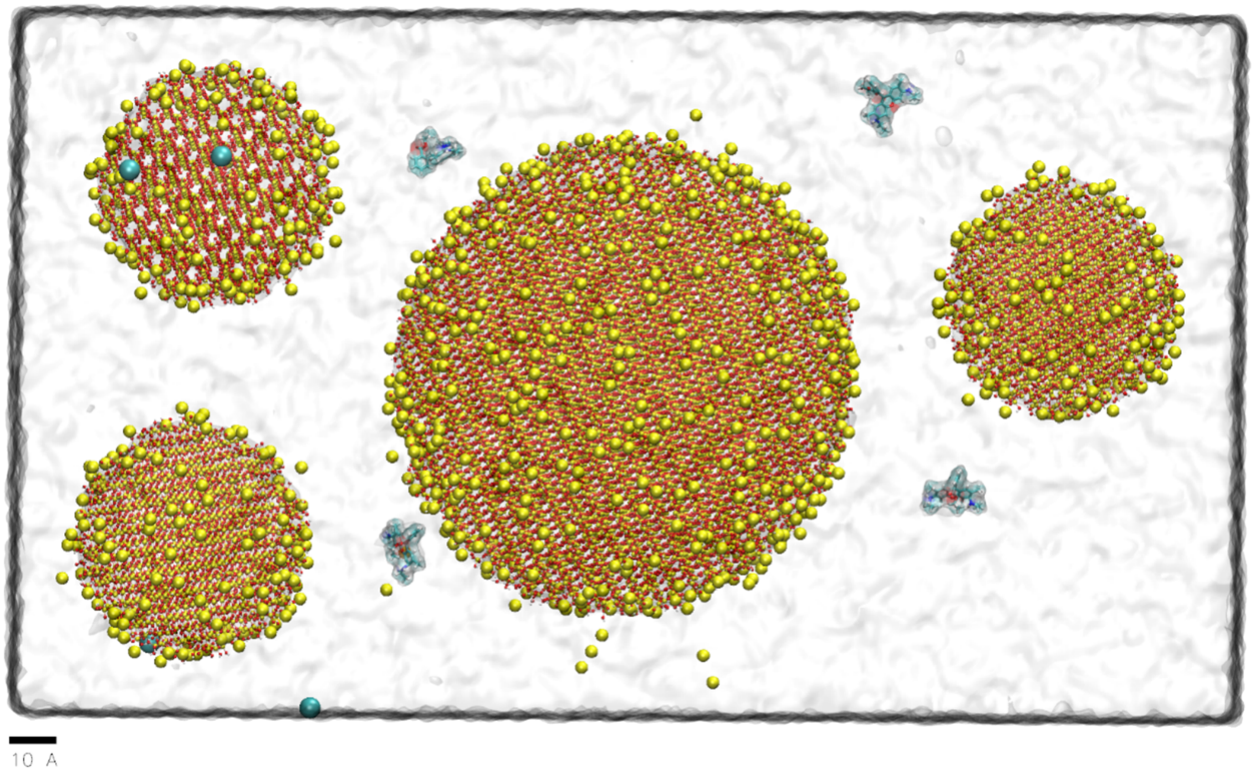
Initial system setup. Water is indicated by the transparent film, the SNPs are represented using CPK representation, ions as vdW spheres, and R6G molecules as liquorice, with the following color coding: oxygen-red, silica-yellow, hydrogen-white, carbon-cyan, chlorine-ice blue, sodium-large yellow spheres.

All simulations were performed using NAMD 3.0 version^41,42^ using the combination of INTERFACE^43,44^ and CHARMM36 FF.^45–47^ As typical in MD simulations the system minimization was a two-step process. First, water-only minimization was performed, consisting of 1000 minimization steps and 100 ps equilibration in 293.15 K. This was followed by the entire system minimization, consisting of 10,000 minimization steps, 30 ps of heating to 293.15 K and 270 ps of thermalization with a 1 fs time step. During the production stage the total length of each trajectory was 100 ns with a time step of 1 fs. For fast and accurate evaluation of electrostatic interactions particle mesh Ewald (PME) was used, while the cutoff for the VdW interactions was set to 12 Å. The analysis utilized a custom center-of-mass (COM) distance measurement script, supplemented by visual inspection using VMD. Since the COM-based method does not account for the conformation of R6G during adsorption, a threshold absorption distance of 10 Å from the SNP surface was chosen.

## Results and Discussion

### Viscosity Measurements and Theoretical Recovery Times

It is essential to have an accurate measurement of the sample viscosity to obtain a precise measurement of particle size distribution. The viscosity of all samples used in this work was therefore measured using a rotational rheometer. The viscosity of HS40, AS40 and their mixtures was measured at 20 °C. The results for the viscosity measurements for the range of samples used in this work are shown in Figure 2.

**Figure 2:**
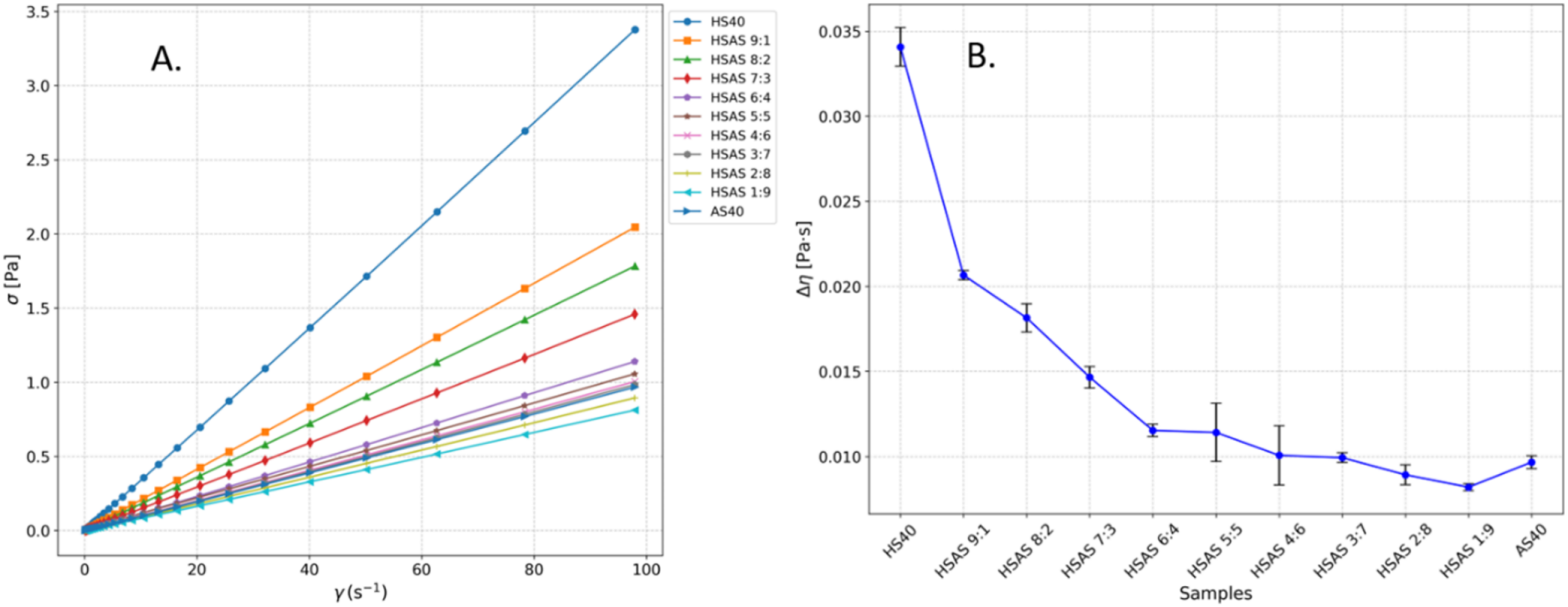
Viscosity Results. (A) Shear stress as a function of shear rate for the used samples; (B) average viscosity used for the size calculations.

Figure 2A shows shear stress as a function of shear rate for each of the samples used. All samples displayed Newtonian behavior over the whole range of measured shear rates.

The viscosities measured between 1 and 100 s^–1^ shear rates were averaged to obtain the viscosity values used for the size calculations. The error bars were taken as three standard deviations of those values. The resulting viscosities are shown in Figure 2B. The measured viscosities were found to be lower than the values specified by the manufacturer for both HS40 and AS40. The measured viscosity of HS40 was 34 cP at 20 °C compared to the manufacturer’s reported 45 cP at 25 °C and 9.6 cP compared to 20 cP for AS40. Only the measured viscosities were used to calculate particle sizes to ensure the consistency of the results between all samples.

The next step was to assess whether the diffusion of each system component occurred on a time scale that could be resolvable by our experimental setup. The theoretical recovery half-times for each sample component—free R6G, AS40 particles, and HS40 particles—are calculated by equating eqs 2 and 3

4 $$\frac{k_{B}T}{6\pi \eta R_{H}}=\Gamma \frac{\omega^{2}}{4\tau }$$

and solving it for τ

5 $$\tau =\Gamma \frac{6\pi \omega^{2}\eta R_{H}}{4k_{B}T}$$

To obtain theoretical recovery times, the radii used were 0.6 nm for R6G,^48,49^ 6 nm for HS40 and 11 nm for AS40, while for the viscosity, the measured values shown in Figure 2B were used. The obtained theoretical recovery half-times are plotted in Figure 3A,B.

**Figure 3:**
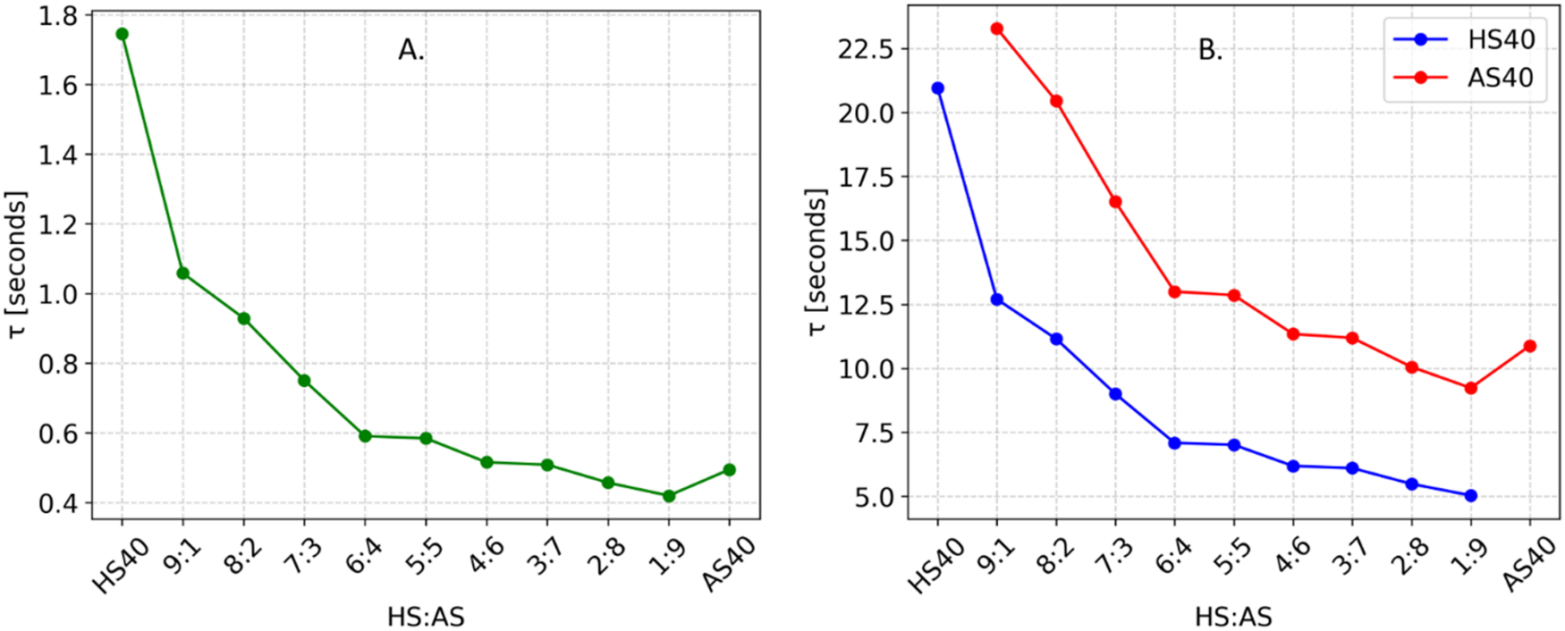
Theoretical recovery half-times for all samples. (A) Free R6G in each sample; (B) HS40 and AS40 colloids.

The R6G recovery half-times shown in Figure 3A indicate that, in most cases, free dye diffuses faster than the measurement limit. However, as shown later, in samples with ratios from 9:1 to 7:3 a model component can still be successfully attributed to free dye, improving radius accuracy when using the more complex triexponential model. In all other samples, adding a third component to describe the kinetics of free R6G would lead to overparametrization and size overestimation. Both colloids are distinguishable across most of the range, but accuracy improves at higher viscosities, where slower overall diffusion allows for a more detailed representation.

### Pure Colloids

Measurements on individual colloids were performed to assess whether the proposed FRAP method can be used successfully to measure the size of nanoparticles in a simple monodisperse system. In both cases of 6 nm HS40 and 11 nm AS40, the dye was added directly to the sample at concentrations ranging from 5 to 20 μM and the experiment was conducted according to the protocol described in the Materials and Methods section. The resulting particle sizes obtained by directly fitting the FRAP recovery curves to both mono and biexponential models are shown in Figure 4.

**Figure 4:**
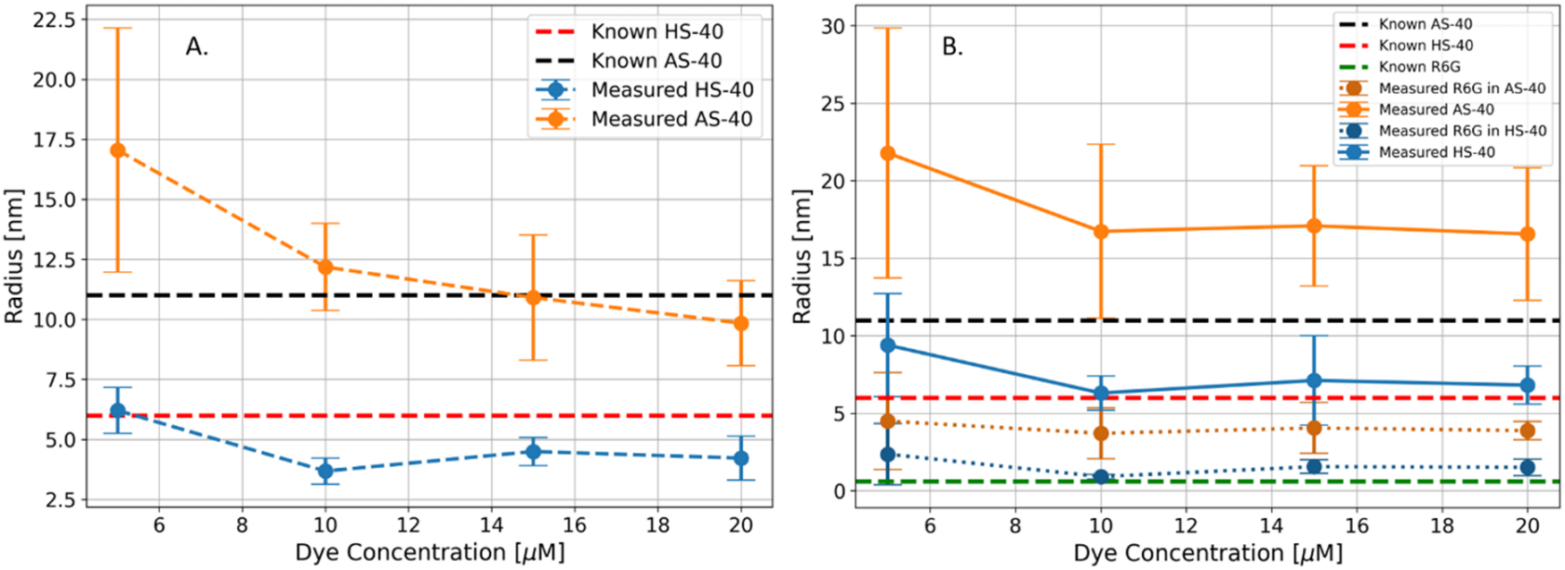
Results for the monodisperse systems: (A) Recovered hydrodynamic radii for LUDOX HS40 and AS40 when fitted to a monoexponential function; (B) recovered hydrodynamic radii for LUDOX HS40 and AS40 when fitted to a biexponential function. All errors are quoted to three standard deviations. The actual sizes for the R6G (0.6 nm) and SNPs (6 nm for HS40 and 11 nm for AS40) are marked with dashed lines.

The results indicate that precise size estimation strongly depends on selecting an appropriate theoretical model based on sample viscosity. The monoexponential model in Figure 3A effectively captures the kinetics of the AS40 colloids and retrieves particle sizes with high precision, particularly at higher dye concentrations. This accuracy can be attributed to the short recovery half-time (0.5 s) of free R6G in AS40 (Figure 3A), which the current experimental setup cannot resolve. Consequently, fully assessing the free dye’s impact on the measured AS40 radius remains challenging. However, the high precision of the recovered sizes suggests that this effect is minimal at the tested concentrations. Nevertheless, the influence of free dye is evident, as shown by the decrease in the average recovered size with increasing dye concentrations in AS40.

In contrast, the monoexponential model fails to accurately recover the radius of HS40, underestimating its size and further emphasizing the dependence of model selection on viscosity. This underestimation arises from free R6G diffusion, which occurs at a partially detectable rate with a recovery half-time of around 2 s. As a result, the oversimplified monoexponential model does not adequately describe free dye diffusion, resulting in a smaller-than-expected size estimate. This effect is evident when comparing the HS40 size at 5 and 20 μM dye concentrations. At 5 μM, where free dye is minimal, the recovered HS40 size matches the reference value of 6 nm. However, at 20 μM, with a substantially higher free dye concentration, the size is underestimated. These findings suggest that in more viscous samples, where both dye and SNP diffusion are observable, a more complex model is necessary.

The recovered hydrodynamic radii (*R*_H_) of both colloids using a biexponential model are shown in Figure 4B. The biexponential model is unsuitable for AS40 because it overparametrizes the experimental data, resulting in significantly overestimated radii for both free R6G and SNP. However, the biexponential model provides a more detailed description of recovery curves in HS40 samples, yielding more accurate hydrodynamic radii. Notably, at higher dye concentrations, the HS40 SNP radius is no longer underestimated, and the recovered R6G radius closely matches the expected value of 0.6 nm.

All of the above suggests that a universal model cannot accurately recover colloidal particle radii. Instead, model selection must be tailored to sample viscosity. Moreover, accounting for free dye is crucial, as oversimplified models tend to underestimate recovered sizes. Further fluorescence lifetime analysis indicated that a free dye population is always detectable, independent of the concentration, however, its relative abundance cannot be accurately quantified across samples based solely on the amplitude. The overall contribution of this component was around 20 to 30%. For detailed analysis refer to Supporting Information.

### Binary Particle Systems

In the previous section, we demonstrated that FRAP can precisely measure the sizes of individual colloids in a monodisperse system. The next step is to determine whether the method can also distinguish individual particle sizes in a binary particle system. To create such a system, LUDOX HS40 and AS40 colloids were mixed at ratios starting from 9:1 (HS40:AS40) to 1:9 ratio, immediately before measurements. The mixtures were added with R6G at concentrations of 5, 10, 15, and 20 μM. FRAP measurements were conducted, and the obtained recovery curves were fitted to multiexponential models as described in the Materials and Methods section. It was found that 10 to 15 μM concentration provided the best balance between the signal-to-noise (S/N) ratio and the measurement precision.

The recovered hydrodynamic radii obtained by fitting the recovery curves to a monoexponential model are shown in Figure 5. These results demonstrated that even the simplest models can provide some insight into sample composition. In samples with high HS40 content, the recovered hydrodynamic radius is close to that of HS40, likely because the 6 nm particles dominate the composition, making them easily identifiable. In contrast, as HS40 content decreases and AS40 concentration increases, the recovered radius shifts closer to the 11 nm AS40 SNPs.

**Figure 5:**
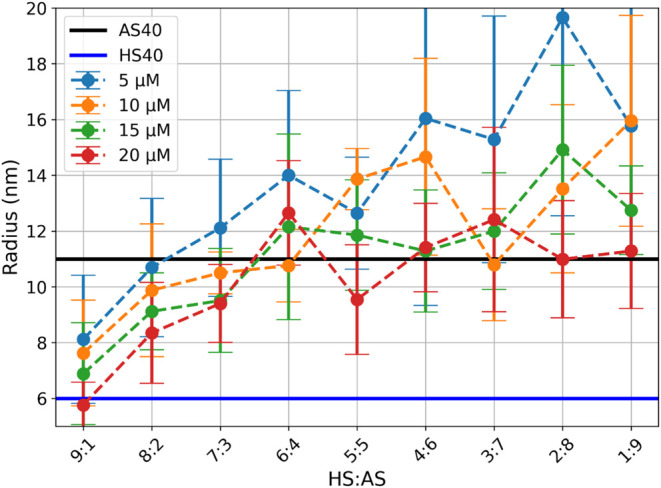
Recovered hydrodynamic radii from fitting the data sets to a monoexponential model for varying dye concentrations.

However, it is essential to highlight that this trend holds only at high dye concentrations. To ensure accurate measurements and reliable recovery of individual component sizes, dye concentration must remain relatively high, around 15–20 μM. Higher dye concentrations improve labeling efficiency, enhance the S/N ratio (as indicated by reduced error bars), and enable accurate size estimation. Additionally, the observed increase in measured radius at lower dye concentrations may result from preferential adsorption of the dye to larger SNPs due to their higher net negative charge and smaller surface curvature. These findings suggest that a simple mixture of two unknown colloids at different ratios can provide sufficient information to estimate particle sizes. However, this approach is suboptimal as accurately determining multiple nanoparticle sizes requires varying their concentrations and conducting at least two measurements. This is impractical, especially for polydisperse, metastable systems such as sodium silicates.^50–52^ Therefore, multiexponential models were tested.

Figure 6 presents the results of fitting the recovery curves to a biexponential model. These results largely mirror the trends observed with the monoexponential model. Specifically, measurement precision for AS40 SNPs improves with increasing dye concentration due to an enhanced S/N ratio, expanding the range of ratios where the size of 11 nm SNP can be accurately measured. However, at higher dye concentrations, the recovered size of the 6 nm HS40 SNP is gradually underestimated, as free dye contributes more to the average measured size of HS40 and R6G. Despite these limitations, the biexponential model significantly outperforms the monoexponential model by successfully identifying the presence of SNPs of different sizes with a single measurement, which the simpler model could not achieve. However, one should note that the biexponential model consistently overestimates the size of the AS40 at all R6G concentrations in samples where the HS40 ratio is 6:4 or lower. Even when AS40 is the dominant component, the recovered size remains overestimated. This reinforces the trend observed in the pure colloid AS40 sample, highlighting viscosity as the key factor limiting measurement precision. Additionally, the detection of 11 nm particles even at very low AS40 concentrations suggests that R6G exhibits size-dependent adsorption, with a greater tendency to bind to larger SNPs.

**Figure 6:**
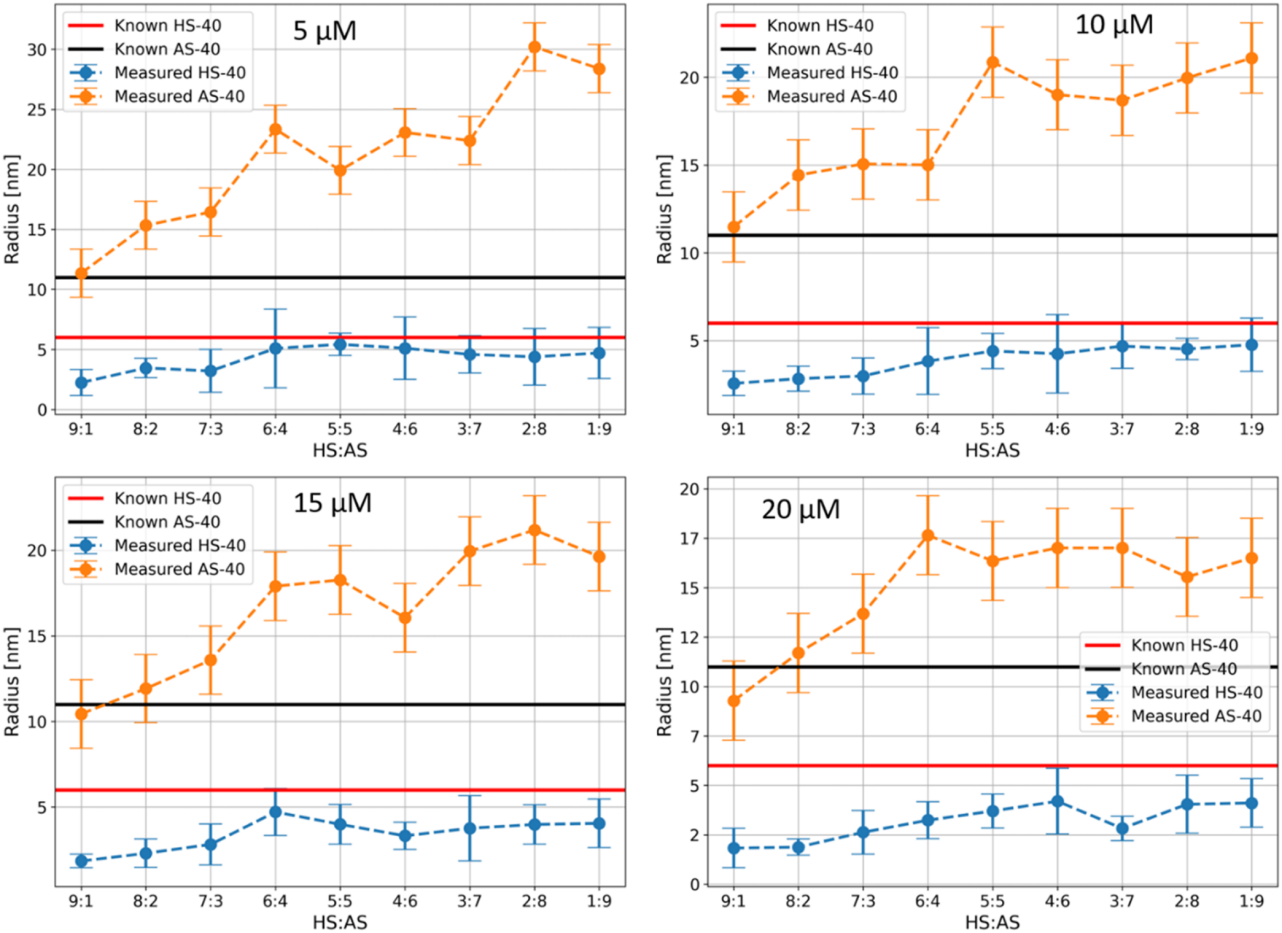
Recovered hydrodynamic radii from fitting the data sets to a biexponential model as a function of dye concentration.

For HS40 SNPs in the mixtures, the recovered size is underestimated in all samples, even when HS40 is the dominant component (as in the 9:1 and 8:2 samples). This likely results from the oversimplified model struggling to accurately describe the diffusion of a three component system. Consequently, the recovered HS40 radius represents a mixture of HS40 and free R6G radii.

Nonetheless, the biexponential model offers the best balance between accuracy and precision, enabling the identification of both larger and smaller SNPs without excessive parametrization while maintaining reasonable accuracy and relatively small error bars. Furthermore, the accuracy of the biexponential model can be improved by performing FRAP measurements based on fluorescence lifetimes^53^ rather than intensity, as lifetime-based measurements would allow for complete separation of free and bound dye to their distinct lifetimes.^5^

To capture all system components within a single measurement a triexponential model was used for fitting GBM preprocessed data, with results shown in Figure 7. As expected, the trends observed in simpler models are also present here. Notably, measurement accuracy improves with increasing dye concentration, as indicated by the significant reduction in error bars when comparing the 9:1 and 8:2 samples at 15 μM versus 5 μM dye concentrations.

**Figure 7:**
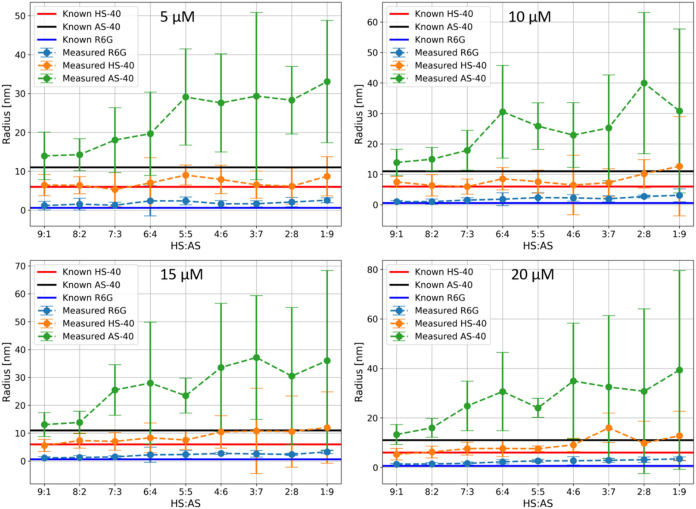
Recovered hydrodynamic radii from fitting the data sets to a triexponential model.

The triexponential model is sufficiently complex to describe the full kinetics of the sample and its components, enabling the recovery of all three sizes in a single measurement at HS40/AS40 ratios of 9:1 and 8:2. However, a slight overestimation of the AS40 SNP radius in the 9:1 and 8:2 samples is likely due to its relatively low concentration in the mixture. HS40, in contrast, can be accurately measured across a broader range of sample ratios due to its higher concentration in the mixture. As the AS40 concentration increases, the overall viscosity rapidly decreases (as shown in Figure 2), leading to faster diffusion. This results in increased variation between FRAP measurement repetitions, larger error bars, and an overestimation of size – an effect also observed in pure AS40 samples (Figure 4B). This is evident in all three components; the recovered size gradually increases with decreasing viscosity, reinforcing the conclusion that optimal model selection strongly depends on sample viscosity and remains a nontrivial process. Nonetheless, at high viscosities, the triexponential model provides a significant advantage over simpler models. It enables useful determination of particle sizes at sufficient accuracy and precision in a binary particle system with a single measurement, even when one colloid is present at low concentrations.

It is important to note that local photobleaching involves high photon fluxes, which can induce transient local heating within the sample. This thermal effect might result in several secondary effects. First, since R6G–colloid binding is relatively weak and reversible, as demonstrated in our previous work,^27^ even modest temperature increases could shift the free/bound equilibrium during bleaching. Subsequent rapid cooling, facilitated by effective thermal diffusion, may trigger dynamic re-equilibration, potentially complicating the interpretation of decay components. Second, temperature-induced changes in viscosity could temporarily enhance dye mobility, thereby affecting the observed recovery kinetics. Third, the degree of local heating may depend on the number of dye molecules adsorbed per silica nanoparticle, potentially introducing size-dependent artifacts. Although these effects are expected to be both localized and transient, they may introduce inconsistencies in the recovered particle sizes and binding ratios, especially when comparing samples with different colloidal compositions.

### Molecular Dynamics Simulations

To assess whether the dye adsorption to the SNP is indeed size and charge-dependent, as indicated by FRAP for the binary particles system, MD simulations on a setup consisting of one large SNP and three small SNPs with varying dye amounts were performed. To reduce computational costs in modeling SNPs with 6 and 11 nm radii, the system was rescaled to 25.7 Å for HS40 and 51.3 Å for AS40 respectively, while the protonation state corresponding to pH 9 and preserving the original SNP size ratio were included. However, system rescaling introduces certain limitations, as it artificially reduces dye-to-SNP adsorption by increasing SNP diffusivity and curvature. As a result, the simulation results should be interpreted as indicative of general trends rather than exact representations of experimental conditions. Notably, in the obtained MD systems, R6G exhibits a 3-fold higher probability of adsorbing to the smaller SNPs compared to larger ones, primarily due to their 3-fold greater abundance. Figure 8 presents the center-of-mass (COM) distance plots as a function of simulation time for each dye-SNP pair in a system containing four dye molecules.

**Figure 8:**
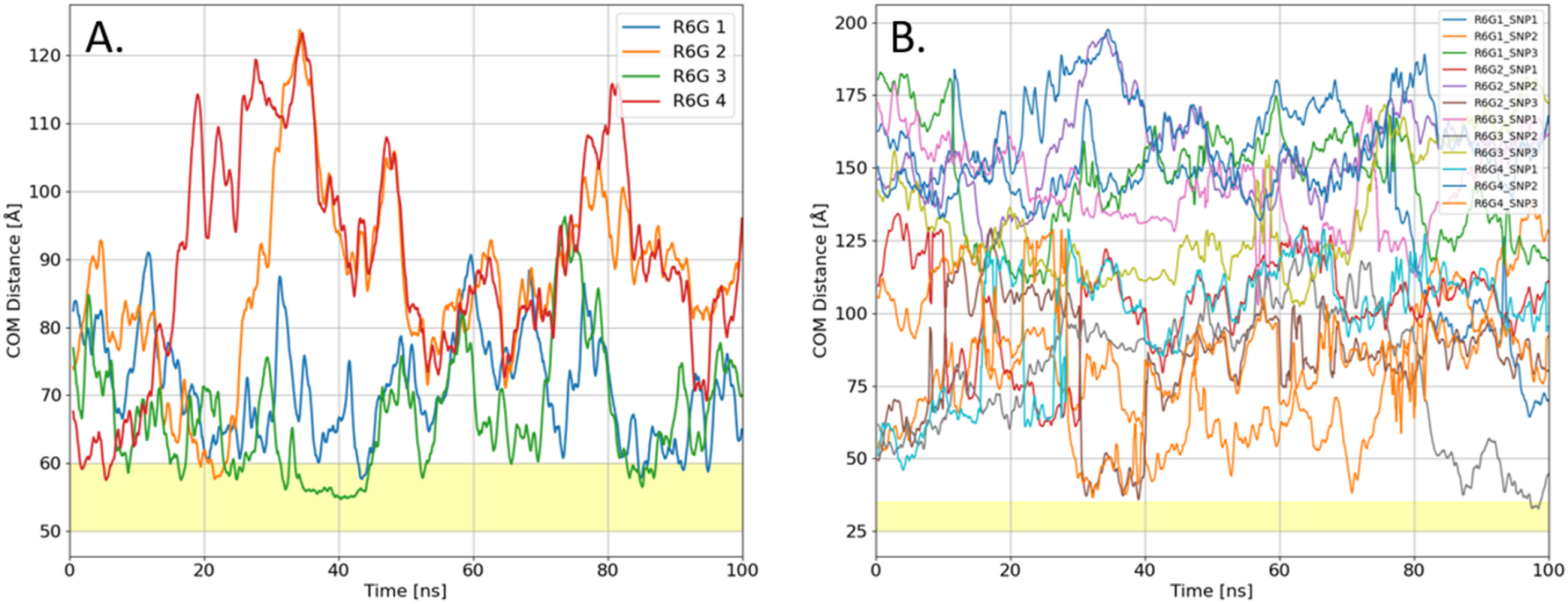
COM distance plots. (A) Each of the four R6G molecules to the 51.3 Å radius SNP; (B) Each R6G-25.7 Å SNP pair. The shaded yellow area marks the range of successful adsorption.

Simulation results indicate that dye adsorption probability and stability are strongly influenced by the radius of the target SNP. It is likely due to the higher net negative charge of the large SNP, which facilitates the localization of favorable absorption sites for the free R6G molecules. Furthermore, the smaller curvature of larger SNPs allows for better shape adjustment for the adsorbing dye, resulting in more stable binding. This is evident in the prolonged period of unchanged COM distance (∼32 to 45 ns) for the large SNP, compared to the much shorter adsorption duration (∼100 ns) for smaller SNPs.

The presence of R6G dimers in the system is also notable and can be identified in the COM distance plots, where overlapping curves of different dyes suggest that two R6G molecules move together. However, as previously discussed in our work,^27^ these dimers do not adsorb onto SNPs and are nonfluorescent,^5^ meaning they have no impact on FRAP measurements. This preferential adsorption of R6G to larger SNP is consistently observed in other systems with fewer dye molecules (data omitted for clarity). Furthermore, in a polydispersed system, the complexity of electrostatic interactions among SNPs of different sizes and dye molecules significantly suppresses adsorption compared to an isolated, monodispersed system.^27^ Despite this suppression, the overall trends remain consistent. In such a system successful adsorption requires R6G molecules to adjust their orientation and conformation in response to the system’s superimposed electric field while maintaining close proximity to an SNP to enable short-range van der Waals interactions to stabilize the SNP-R6G complex.

In summary, MD simulations demonstrate that R6G adsorption rates are highly dependent on SNP size, with larger SNPs promoting stronger binding. This is largely attributed to their greater net negative charge and smaller curvature, which facilitate dye conformation adjustment during adsorption. A similar trend was observed in FRAP experiments on binary particle systems, where lower dye concentrations allowed for more precise measurement of larger SNPs. MD simulations provide valuable insight into this phenomenon.

## Conclusions

In this work, the use of FRAP as a nanometrology tool, supported by MD simulations, in binary nanoparticle systems was assessed for the first time. Unlike our previous studies using time-resolved anisotropy measurements which only provide an average particle size for a two-particle system, FRAP enabled the recovery of individual particle sizes. However, data analysis is nontrivial, as model selection is highly dependent on the sample viscosity. Our results indicate that highly viscous samples, containing slowly diffusing particles, benefit from more complex models, whereas simpler models are more suitable for less viscous systems to avoid overparametrization and size overestimation. This suggests a general trend: multiexponential models are preferable for high-viscosity samples with slow diffusion, while simpler models are more effective for thinner fluids where diffusion occurs rapidly. Furthermore, optimal dye concentration was found to be around 15 μM, ensuring a strong S/N ratio. However, high dye concentrations introduces significant amounts of free dye, which can lead to underestimated sizes due to size mixing between nanoparticles and unbound dye. This issue could be mitigated by using a fluorescent dye that forms a covalent siloxane bond,^54,55^ reducing free dye presence and improving measurement precision. Alternatively, FRAP measurements based on fluorescence lifetime^53^ rather than intensity could completely separate signals from free and bound dye, as our previous work demonstrated distinct lifetimes for each.^27^

Regardless of the model used, our findings confirm that FRAP is a viable nanometrology tool for measuring SNP sizes. Even the simplest monoexponential model provides size estimates with just two measurements. The most precise size estimate was achieved using the triexponential model, where each component corresponds to a distinct sample fraction. However, this model is only reliable in relatively high-viscosity environments, as it tends to underperform at low viscosities, leading to size overestimation and overparametrization. Finally, MD simulations revealed that dye adsorption is size-dependent, with larger SNPs serving as more suitable targets. This is largely due to their greater net negative charge and smaller curvature, which facilitate more stable dye adsorption. These observations align with trends observed in FRAP experiments, reinforcing the potential of combining both methods to enhance our understanding of binary nanoparticle systems.

When prior knowledge of their binary nature exists, the biexponential model offers the best balance between accuracy, simplicity and reliability for binary particle systems. While it slightly underestimates the size of smaller nanoparticles, its advantages outweigh its drawbacks. The combination of covalently bound dye at lower concentrations with lifetime-based FRAP should potentially further enhance measurement accuracy, enabling precise size determination for both nanoparticle species in the system.

## Supplementary Material



## Data Availability

Data are contained within the article. Any additional data needed will be shared on a reasonable request to the corresponding author.

## Notes

Detailed fluorescence intensity decay analyses, including plotted raw decays and fitting parameters (PDF)

## References

[1] Bhushan, B. Introduction to Nanotechnology. In Springer Handbook of Nanotechnology; Springer Berlin Heidelberg: Berlin, Heidelberg, 2017; pp 1–19.

[2] Williams, D. B. ; Carter, C. B. The Transmission Electron Microscope; Springer, 1996.

[3] Pauw B. R. (2013). Everything
SAXS: small-angle scattering pattern collection and correction. J. Phys.: Condens. Matter.

[4] Uskoković V. (2012). Dynamic light
scattering based microelectrophoresis: main prospects and limitations. J. Dispersion Sci. Technol..

[5] Doveiko D., Martin A. R. G., Vyshemirsky V., Stebbing S., Kubiak-Ossowska K., Rolinski O., Birch D. J. S., Chen Y. (2024). Nanoparticle Metrology
of Silicates Using Time-Resolved Multiplexed Dye Fluorescence Anisotropy,
Small Angle X-ray Scattering, and Molecular Dynamics Simulations. Materials.

[6] Birch D. J. S., Geddes C. D. (2000). Sol-gel particle growth studied using fluorescence
anisotropy: An alternative to scattering techniques. Phys. Rev. E.

[7] Karolin J., Geddes C. D., Wynne K., Birch D. J. S. (2002). Nanoparticle
metrology in sol-gels using multiphoton excited fluorescence. Meas. Sci. Technol..

[8] Chalfie M., Tu Y., Euskirchen G., Ward W. W., Prasher D. C. (1994). Green fluorescent
protein as a marker for gene expression. Science.

[9] Reits E. A., Neefjes J. J. (2001). From fixed to FRAP: measuring protein mobility and
activity in living cells. Nat. Cell Biol..

[10] Lorén N., Hagman J., Jonasson J. K., Deschout H., Bernin D., Cella-Zanacchi F., Diaspro A., McNally J. G., Ameloot M., Smisdom N., Nydén M., Hermansson A.-M., Rudemo M., Braeckmans K. (2015). Fluorescence
recovery after photobleaching
in material and life sciences: putting theory into practice. Q. Rev. Biophys..

[11] Axelrod D., Koppel D. E., Schlessinger J., Elson E., Webb W. W. (1976). Mobility
measurement by analysis of fluorescence photobleaching recovery kinetics. Biophys. J..

[12] Deschout H., Hagman J., Fransson S., Jonasson J., Rudemo M., Lorén N., Braeckmans K. (2010). Straightforward FRAP for quantitative
diffusion measurements with a laser scanning microscope. Opt. Express.

[13] Singer J. R. (1978). NMR diffusion
and flow measurements and an introduction to spin phase graphing. J. Phys. E: Sci. Instrum..

[14] Bernin D., Topgaard D. (2013). NMR diffusion and relaxation
correlation methods: New
insights in heterogeneous materials. Curr. Opin.
Colloid Interface Sci..

[15] Liebman P. A., Entine G. (1974). Lateral diffusion of
visual pigment in photoreceptor
disk membranes. Science.

[16] Sprague B. L., Pego R. L., Stavreva D. A., McNally J. G. (2004). Analysis of Binding
Reactions by Fluorescence Recovery after Photobleaching. Biophys. J..

[17] Stenoien D. L., Patel K., Mancini M. G., Dutertre M., Smith C. L., O’Malley B. W., Mancini M. A. (2001). FRAP reveals that mobility of oestrogen
receptor-α is ligand-and proteasome-dependent. Nat. Cell Biol..

[18] Bulinski J. C., Odde D. J., Howell B. J., Salmon T. D., Waterman-Storer C. M. (2001). Rapid dynamics
of the microtubule binding of ensconsin in vivo. J. Cell Sci..

[19] Sarkar, P. ; Chattopadhyay, A. Exploring Membrane Lipid and Protein Diffusion by FRAP. In Analysis of Membrane Lipids; Prasad, R. ; Singh, A. , Eds.; Springer US: New York, NY, 2020; pp 119–141.

[20] Mueller F., Mazza D., Stasevich T. J., McNally J. G. (2010). FRAP and kinetic
modeling in the analysis of nuclear protein dynamics: what do we really
know?. Curr. Opin. Cell Biol..

[21] Giakoumakis, N. N. ; Rapsomaniki, M. A. ; Lygerou, Z. Analysis of Protein Kinetics Using Fluorescence Recovery after Photobleaching (FRAP). In Methods in Molecular Biology; Springer, 2017; Vol. 1563, pp 243–267. 28324613 10.1007/978-1-4939-6810-7_16

[22] Xia Y., Wu J., Wei W., Du Y., Wan T., Ma X., An W., Guo A., Miao C., Yue H., Li S., Cao X., Su Z., Ma G. (2018). Exploiting the pliability
and lateral
mobility of Pickering emulsion for enhanced vaccination. Nat. Mater..

[23] Lee D., Weitz D. A. (2008). Double emulsion-templated nanoparticle colloidosomes
with selective permeability. Adv. Mater..

[24] Singh V. K., Yadav I., Kulanthaivel S., Roy B., Giri S., Maiti T. K., Banerjee I., Pal K. (2016). Groundnut oil based
emulsion gels for passive and iontophoretic delivery of therapeutics. Des. Monomers Polym..

[25] Brake J. M., Daschner M. K., Abbott N. L. (2005). Formation
and Characterization of
Phospholipid Monolayers Spontaneously Assembled at Interfaces between
Aqueous Phases and Thermotropic Liquid Crystals. Langmuir.

[26] Pihl M., Kolman K., Lotsari A., Ivarsson M., Schüster E., Lorén N., Bordes R. (2019). Silica-based diffusion probes for
use in FRAP and NMR-diffusometry. J. Dispersion
Sci. Technol..

[27] Doveiko D., Kubiak-Ossowska K., Chen Y. (2024). Impact of the Crystal
Structure of
Silica Nanoparticles on Rhodamine 6G Adsorption: A Molecular Dynamics
Study. ACS Omega.

[28] Schneider C. A., Rasband W. S., Eliceiri K. W. (2012). NIH Image to ImageJ:
25 years of
image analysis. Nat. Methods.

[29] Abràmoff M. D., Magalhães P. J., Ram S. J. (2004). Image processing with ImageJ. Biophotonics Int..

[30] Van Rossum, G. ; Drake, F. L. Python Reference Manual; Centrum voor Wiskunde en Informatica: Amsterdam, 1995; Vol. 111.

[31] Harris C. R., Millman K. J., Van Der
Walt S. J., Gommers R., Virtanen P., Cournapeau D., Wieser E., Taylor J., Berg S., Smith N. J. (2020). Array programming with NumPy. Nature.

[32] Friedman J. H. (2001). Greedy
function approximation: a gradient boosting machine. Ann. Stat..

[33] Hastie, T. ; Tibshirani, R. ; Friedman, J. Boosting and Additive Trees. In Elements of Statistical Learning: Data Mining, Inference, and Prediction; Springer, 2009; pp 337–387.

[34] Jo S., Kim T., Iyer V. G., Im W. (2008). CHARMM-GUI: a web-based graphical
user interface for CHARMM. J. Comput. Chem..

[35] Choi Y. K., Kern N. R., Kim S., Kanhaiya K., Afshar Y., Jeon S. H., Jo S., Brooks B. R., Lee J., Tadmor E. B. (2022). CHARMM-GUI
nanomaterial modeler for modeling
and simulation of nanomaterial systems. J. Chem.
Theory Comput..

[36] Chuichay P., Vladimirov E., Siriwong K., Hannongbua S., Rösch N. (2006). Molecular-dynamics simulations of pyronine 6G and rhodamine
6G dimers in aqueous solution. J. Mol. Model..

[37] Vaiana A. C., Schulz A., Wolfrum J., Sauer M., Smith J. C. (2003). Molecular
mechanics force field parameterization of the fluorescent probe rhodamine
6G using automated frequency matching. J. Comput.
Chem..

[38] Kern N. R., Lee J., Choi Y. K., Im W. (2024). CHARMM-GUI Multicomponent Assembler
for modeling and simulation of complex multicomponent systems. Nat. Commun..

[39] Humphrey W., Dalke A., Schulten K. (1996). VMD: visual molecular dynamics. J. Mol. Graphics.

[40] Mark P., Nilsson L. (2001). Structure and dynamics
of the TIP3P, SPC, and SPC/E
water models at 298 K. J. Phys. Chem. A.

[41] Phillips J.
C., Braun R., Wang W., Gumbart J., Tajkhorshid E., Villa E., Chipot C., Skeel R. D., Kale L., Schulten K. (2005). Scalable molecular dynamics with NAMD. J. Comput. Chem..

[42] Phillips J. C., Hardy D. J., Maia J. D., Stone J. E., Ribeiro J. V., Bernardi R. C., Buch R., Fiorin G., Hénin J., Jiang W. (2020). Scalable
molecular dynamics on CPU and GPU architectures
with NAMD. J. Chem. Phys..

[43] Heinz H., Lin T.-J., Mishra R. K., Emami F. S. (2013). Thermodynamically
Consistent Force Fields for the Assembly of Inorganic, Organic, and
Biological Nanostructures: The INTERFACE Force Field. Langmuir.

[44] Heinz H., Vaia R. A., Farmer B. L., Naik R. R. (2008). Accurate Simulation
of Surfaces and Interfaces of Face-Centered Cubic Metals Using 12–6
and 9–6 Lennard-Jones Potentials. J.
Phys. Chem. C.

[45] Huang J., MacKerell A. D. (2013). CHARMM36 all-atom additive protein
force field: Validation based on comparison to NMR data. J. Comput. Chem..

[46] Huang J., Rauscher S., Nawrocki G., Ran T., Feig M., de Groot B. L., Grubmüller H., MacKerell A. D. (2017). CHARMM36m:
an improved force field for folded and intrinsically disordered proteins. Nat. Methods.

[47] Vanommeslaeghe K., Hatcher E., Acharya C., Kundu S., Zhong S., Shim J., Darian E., Guvench O., Lopes P., Vorobyov I., Mackerell A. D. (2010). CHARMM general force field: A force
field for drug-like molecules compatible with the CHARMM all-atom
additive biological force fields. J. Comput.
Chem..

[48] Müller C. B., Loman A., Pacheco V., Koberling F., Willbold D., Richtering W., Enderlein J. (2008). Precise measurement
of diffusion by multi-color dual-focus fluorescence correlation spectroscopy. Europhys. Lett..

[49] Gumy J.-C., Vauthey E. (1996). Picosecond polarization
grating study of the effect
of excess excitation energy on the rotational dynamics of rhodamine
6G in different electronic states. J. Phys.
Chem. A.

[50] Matinfar M., Nychka J. A. (2023). A review of sodium silicate solutions: Structure, gelation,
and syneresis. Adv. Colloid Interface Sci..

[51] Nordström J., Sundblom A., Jensen G. V., Pedersen J. S., Palmqvist A., Matic A. (2013). Silica/alkali ratio
dependence of the microscopic structure of sodium
silicate solutions. J. Colloid Interface Sci..

[52] Hu G., Jin S., Liu K. (2023). Structure-Directing
Effect on Silica Nanoparticle Growth
in Sodium Silicate Solutions through Small-Angle X-ray Scattering. J. Phys. Chem. C.

[53] Mori I., Terasaka S., Yamaguchi S., Otosu T. (2024). Diffusion of Multiple
Species Resolved by Fluorescence Lifetime Recovery after Photobleaching
(FLRAP). Anal. Chem..

[54] Stewart H. L., Yip P., Rosenberg M., Sørensen T. J., Laursen B. W., Knight A. E., Birch D. J. S. (2016). Nanoparticle
metrology of silica colloids and super-resolution
studies using the ADOTA fluorophore. Meas. Sci.
Technol..

[55] Tleugabulova D., Zhang Z., Chen Y., Brook M. A., Brennan J. D. (2004). Fluorescence
Anisotropy in Studies of Solute Interactions with Covalently Modified
Colloidal Silica Nanoparticles. Langmuir.

