# Supplementary material for: Quantitative Nanometrology of Binary Particle Systems Using Fluorescence Recovery after Photobleaching: Application to Colloidal Silica
Source: Langmuir. 2025 May 19;41(29):19173–82. doi: 10.1021/acs.langmuir.5c01287 (PMC12312185; doi:10.1021/acs.langmuir.5c01287)
Supplement: Supplementary file 1 [file la5c01287_si_001.pdf]

Supporting Information

Quantitative Nanometrology of Binary Particle Systems Using Fluorescence Recovery After Photobleaching: Application to Colloidal Silica

Daniel Doveiko[1*], Lisa Asciak[2], Simon Stebbing[3], Wenmiao Shu[2], Karina Kubiak-Ossowska[4], David J.S. Birch[1] and Yu Chen[1*]

[1] Photophysics Group, Department of Physics, University of Strathclyde, Glasgow G4 0NG, UK
[2] Department of Biomedical Engineering, University of Strathclyde, Glasgow G4 0NW, UK
[3] PQ Silicas UK Limited, Warrington WA5 1AB, UK
[4] ARCHIE-WeSt, Department of Physics, University of Strathclyde, Glasgow G4 0NG, UK
*Corresponding authors: daniel.doveiko.2018@uni.strath.ac.uk; y.chen@strath.ac.uk

Content:

Supplementary Materials: Time-Correlated Single-Photon Counting (TCSPC) Experiments

1. **Methods**
   Detailed description of the experimental setup, instrumentation, and procedures used in the TCSPC measurements.

2. **Results and Discussion:**
   a. Raw Decay Curves
      Presentation of the unprocessed photon decay data collected during the experiments
   b. Fitted Parameters
      Summary of the fitting results, including lifetimes and associated fitting statistics.
   c. Interpretation and Analysis
      Discussion of the implications of the results and insights derived from the data

Supplementary Materials

Time-Correlated Single-Photon Counting Experiments

Methods

Fluorescence intensity decay measurements to obtain lifetime data were based on the time-correlated single-photon counting (TCSPC) [1] technique and performed using a HORIBA-IBH (Glasgow, UK) DeltaFlex system with both excitation and emission Seya-Namioka monochromators which incorporate holographic diffraction gratings to minimise the detection of the scattered light. The R6G-labelled LUDOX colloids were excited using a HORIBA-IBH NanoLED with a centre wavelength of 494 nm, a pulse duration of 1.5 ns and a repetition rate of 1MHz. The emission was collected at 548 nm. Fluorescence decay data were collected using the FluoroHub-A electronics containing a time-to-amplitude (TAC) converter, with the start-to-stop rate kept below 1% to minimise data pile-up [1]. The individual fluorescence decays were measured at a magic angle 54.7° to eliminate orientational effects [1]. Data analysis was carried out using HORIBA DAS6 software.

Results and Discussion

Time-resolved fluorescence measurements were performed on Rhodamine 6G (R6G) in aqueous colloidal silica suspensions composed of HS40 and AS40 Ludox and their mixtures in varying ratios. Measurements were performed at four dye concentrations (5, 10, 15, and 20 µM). The raw fluorescence intensity decays of different samples labelled with R6G are shown in Figure S1.

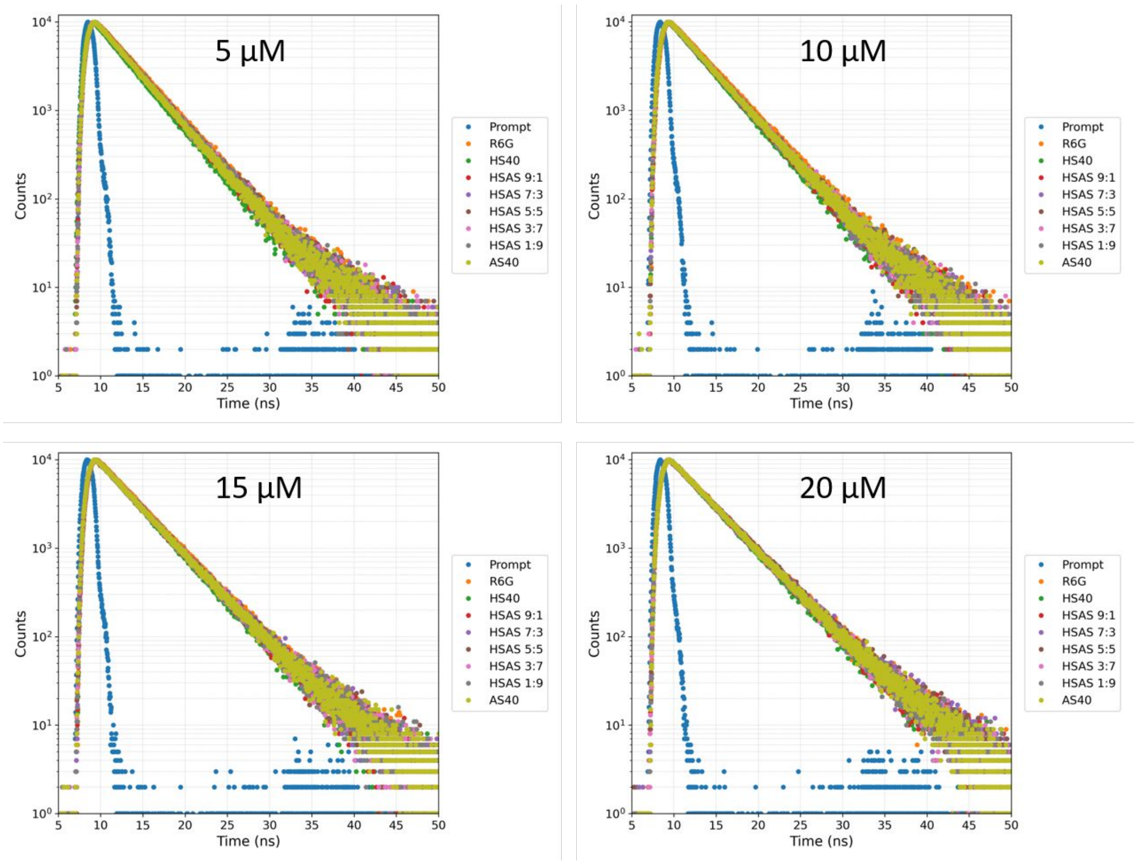

Figure S1 Raw fluorescence intensity decays of Rhodamine 6G (R6G) at 5, 10, 15, and 20 µM in different samples. The samples include pure Ludox HS40, pure Ludox AS40, and their mixtures at various volume ratios (HSAS 9:1 to 1:9). "Prompt" represents the instrument response function.

To obtain the corresponding lifetimes, the decays were fitted to a multi-exponential function:

$$I(t) = \sum_i^n B_i(t) exp\left[-\frac{t}{T_i}\right] \qquad (S1)$$

Decay profiles in colloid-containing samples were best described by bi-exponential functions, indicating the presence of two distinct dye populations: one free in solution and the other adsorbed onto colloid surfaces. The recovered fitting parameters are shown in Tables 1 to 4.

Table S1 Fitting results for the 5 µM samples. T_1 and T_2 are fluorescence lifetimes in nanoseconds (ns), B_1 and B_2 are their respective amplitude contributions in percent (%). The χ^2 value indicates the goodness of fit for each decay curve. The errors are quoted to three standard deviations. The ratio (9 to 1 etc.), corresponds to HS40 to AS40 mixture.

Sample	T_1 (ns)	B_1 (%)	T_2 (ns)	B_2 (%)	X^2
HS40	2.76±0.68	18.31	4.05±0.05	81.69	1.11
9 to 1	3.04±0.68	22.72	4.15±0.06	77.28	1.12
7 to 3	3.19±0.64	27.45	4.25±0.07	72.55	1.10
5 to 5	3.32±0.51	35.46	4.37±0.08	64.54	1.11
3 to 7	3.25±0.71	29.08	4.25±0.07	70.92	1.04
1 to 9	3.26±0.66	27.89	4.24±0.07	72.11	1.04
AS40	3.46±0.64	48.49	4.32±0.12	51.51	1.06
R6G	4.07±0.10	100	[redacted]		1.13

Table S2 Fitting results for the 10 µM samples. T_1 and T_2 are fluorescence lifetimes in nanoseconds (ns), B_1 and B_2 are their respective amplitude contributions in percent (%). The χ^2 value indicates the goodness of fit for each decay curve. The errors are quoted to three standard deviations. The ratio (9 to 1 etc.), corresponds to HS40 to AS40 mixture.

Sample	T_1 (ns)	B_1 (%)	T_2 (ns)	B_2 (%)	X^2
HS40	2.86±0.68	16.71	4.16±0.04	83.29	1.05
9 to 1	2.93±0.73	15.19	4.23±0.04	84.81	1.11
7 to 3	3.33±0.54	25.49	4.36±0.06	74.51	1.06
5 to 5	3.51±0.65	31.81	4.46±0.08	68.19	1.04
3 to 7	3.25±0.81	20.56	4.32±0.06	79.44	1.06
1 to 9	3.35±0.92	26.77	4.36±0.07	73.23	1.13
AS40	3.34±0.50	25.48	4.34±0.07	74.52	1.04
R6G	4.22±0.10	100	[redacted]		1.09

Table S3 Fitting results for the 15 µM samples. T_1 and T_2 are fluorescence lifetimes in nanoseconds (ns), B_1 and B_2 are their respective amplitude contributions in percent (%). The χ^2 value indicates the goodness of fit for each decay curve. The errors are quoted to three standard deviations. The ratio (9 to 1 etc.), corresponds to HS40 to AS40 mixture.

Sample	T_1 (ns)	B_1 (%)	T_2 (ns)	B_2 (%)	X^2
HS40	3.05±0.70	15.32	4.30±0.05	84.68	1.06
9 to 1	2.95±0.87	10.29	4.35±0.04	89.71	1.04
7 to 3	3.48±0.68	21.15	4.50±0.07	78.85	1.04
5 to 5	3.37±0.86	18.53	4.47±0.06	81.47	1.08
3 to 7	3.34±0.66	19.46	4.45±0.06	80.54	1.04
1 to 9	3.42±0.63	23.90	4.48±0.07	76.1	1.06
AS40	3.35±0.83	19.37	4.44±0.06	80.63	1.10
R6G	4.33±0.10	100	[redacted]		1.10

Table S4 Fitting results for the 20 µM samples. T_1 and T_2 are fluorescence lifetimes in nanoseconds (ns), B_1 and B_2 are their respective amplitude contributions in percent (%). The χ^2 value indicates the goodness of fit for each decay curve. The errors are quoted to three standard deviations. The ratio (9 to 1 etc.), corresponds to HS40 to AS40 mixture.

Sample	T_1 (ns)	B_1 (%)	T_2 (ns)	B_2 (%)	X^2
HS40	3.32±0.76	20.86	4.51±0.06	79.14	1.09
9 to 1	3.20±0.83	12.90	4.55±0.05	87.1	1.13
7 to 3	3.47±0.90	17.62	4.64±0.06	82.38	1.12
5 to 5	3.62±0.71	23.77	4.66±0.07	76.23	1.06
3 to 7	3.54±0.74	21.60	4.58±0.07	78.4	0.99
1 to 9	3.61±0.73	27.32	4.64±0.07	72.68	1.01
AS40	3.64±0.41	29.24	4.66±0.07	70.76	1.07
R6G	4.44±0.10	100	[redacted]		1.18

The longer lifetime component (T_2~4.1–4.6 ns) corresponds to dye molecules adsorbed on the colloidal nanoparticle surface, where restricted motion and local stabilisation reduce non-radiative decay [2]. The shorter lifetime component (T_1 ~2.8–3.6 ns) corresponds to free R6G in solution. This component is notably shorter than the lifetime observed in water solutions without colloids (~4.1–4.4 ns), which is consistent with literature values for R6G in neutral environments. The decrease in the free dye's lifetime is attributed to the elevated pH (~9) of the colloidal suspensions, which is known to enhance non-radiative decay pathways in R6G and lead to lifetime quenching [3, 4]. This was previously reported in the literature and is further confirmed by our recent findings [3, 4].

With increasing dye concentration in the samples, T_2 shows a modest increase, which may reflect dye-dye interactions on the colloid, or inner filtering effects due to elevated dye concentration in the solution. However, B_1, which is the amplitude associated with free dye, remains present in all samples and does not change significantly, nor exhibit any consistent trends, which might correspond with the changes in the dye concentration in the sample. This indicates, that a free dye population is always detectable, however, its relative abundance cannot be precisely quantified across samples based solely on the amplitude. Nonetheless, the presence of a shortened T_1 component across all colloid-containing systems confirms that free dye is consistently affected by the basic environment, while the adsorption of the dye to the nanoparticle surface appears to provide a stabilising environment.

Overall, the analysis demonstrates that the colloid type and mixing ratio substantially influence the adsorption behaviour of R6G, while the total dye concentration has a more modest effect on the fluorescence decay parameters.

1. Birch, D. J.; Imhof, R. E., Time-domain fluorescence spectroscopy using time-correlated single-photon counting. In *Topics in Fluorescence Spectroscopy: Techniques*, Springer: 1991; pp 1-95.
2. Lakowicz, J. R., *Principles of fluorescence spectroscopy*. Springer: 2006.
3. Doveiko, D.; Martin, A. R.; Vyshemirsky, V.; Stebbing, S.; Kubiak-Ossowska, K.; Rolinski, O.; Birch, D. J.; Chen, Y., Nanoparticle Metrology of Silicates Using Time-Resolved Multiplexed Dye Fluorescence Anisotropy, Small Angle X-ray Scattering, and Molecular Dynamics Simulations. *Materials* **2024,** *17* (7), 1686.
4. Haimerl, J. M.; Ghosh, I.; König, B.; Lupton, J. M.; Vogelsang, J., Chemical photocatalysis with rhodamine 6g: investigation of photoreduction by simultaneous fluorescence correlation spectroscopy and fluorescence lifetime measurements. *The Journal of Physical Chemistry B* **2018,** *122* (47), 10728-10735.
